# Host Cell Oxidative Stress Induces Dormant Staphylococcus aureus Persisters

**DOI:** 10.1128/spectrum.02313-21

**Published:** 2022-02-23

**Authors:** Frédéric Peyrusson, Tiep Khac Nguyen, Tome Najdovski, Françoise Van Bambeke

**Affiliations:** a Pharmacologie cellulaire et moléculaire, Louvain Drug Research Institute, Université catholique de Louvain (UCLouvain), Brussels, Belgium; b Croix-Rouge de Belgique, Suarlée, Belgium; University of Pittsburgh

**Keywords:** *Staphylococcus aureus*, macrophages, intracellular infection, oxidative stress, persisters, dormancy

## Abstract

Persisters are transiently nongrowing and antibiotic-tolerant phenotypic variants identified in major human pathogens, including intracellular Staphylococcus aureus. Due to their capacity to regrow once the environmental stress is relieved and to promote resistance, persisters possibly contribute to therapeutic failures. While persistence and its related quiescence have been mostly studied under starvation, little is known within host cell environments. Here, we examined how the level of reactive oxygen species (ROS) in different host cells affects dormancy depth of intracellular S. aureus. Using single-cell approaches, we found that host ROS induce variable dormant states in S. aureus persisters, displaying heterogeneous and increased lag times for resuscitation in liquid medium. Dormant persisters displayed decreased translation and energy metabolism, but remained infectious, exiting from dormancy and resuming growth when reinoculated in low-oxidative-stress cells. In high-oxidative-stress cells, ROS-induced ATP depletion was associated with the formation of visible dark foci similar to those induced by the protein aggregation inducer CCCP (carbonyl cyanide *m*-chlorophenylhydrazone) and with the recruitment of the DnaK-ClpB chaperone system involved in the clearance of protein aggregates. ATP depletion led to higher fractions of dormant persisters than ROS, due to a counterbalancing effect of ROS-induced translational repression, suggesting a pivotal role of translation in the dormant phenotype. Consistently, protein synthesis inhibition limited dormancy to levels similar to those observed in low-oxidative-stress cells. This study supports the hypothesis that intracellular S. aureus persisters can reach heterogeneous dormancy depths and highlights the link between ROS, ATP depletion, dark focus formation, and subsequent dormancy state.

**IMPORTANCE** By their capacity to survive to antibiotic pressure and to regrow and give rise to a susceptible population once this pressure is relieved, intracellular persisters of S. aureus may contribute to explain therapeutic failures and recurrent infections. Here, we show that the level of dormancy and the subsequent capacity to resuscitate from this resting state are dependent on the level of oxidative stress in the host cells where bacteria survive. This observation nourishes the debate as whether the most appropriate strategy to cope with S. aureus intracellular infections would consist of trying to push persisters to a deep dormancy state from which wakening is improbable or, on the contrary, to prevent ROS-induced dormancy and force bacteria to maintain regular metabolism in order to restore their responsiveness to antibiotics. Importantly also, our data highlight the interest in single-cell analyses with conventional enumeration of CFU to quantify persisters and study host-pathogen interactions.

## INTRODUCTION

Persisters are nonreplicative and antibiotic-tolerant subpopulations within an isogenic population (1, 2). In fluctuating environments, they may follow different evolutionary pathways, either by reverting to replicative forms or by acquiring resistance, which likely contributes to relapsing infections (3, 4). However, only a few studies have examined their behavior in complex environments such as eukaryotic cells (5, 6).

Besides stochastic generation in bacterial populations, persisters can occur in response to environmental cues, including nutrient starvation, antibiotics, intracellular environment, or oxidative stress (6–10), leading to antibiotic tolerance (11), metabolic quiescence, and decreased proliferation rates (12).

Closely related to persistence, another notion concerns dormancy, which rather defines a reversible state of low metabolic activity, in which bacteria can persist for prolonged periods of time without division (2, 13)—generally associated with low ATP content or protein synthesis (14, 15). The concept of dormancy depth among persisters has been suggested by the team of Zhang et al., who proposed the existence of “shallow” and “deep” persisters (16). Since then, dormancy depth of persisters has been experimentally confirmed in planktonic Escherichia coli cells (17, 18). During starvation, it has been shown that bacterial cells fail to maintain regular metabolism. The subsequent ATP depletion promotes protein aggregation, leading to increased lag time for cell resuscitation, correlated with the bacterial dormancy depth (17). Other phenotypes of dormant bacteria have been described, like the viable but non-culturable (VBNC) phenotype, in which cells remain viable, but in contrast to persisters, do not resume growth in medium on which they normally actively divide, unless specifically stimulated (13, 19–21). Because of the similarities between persisters and VBNC cells, these phenotypes are described as part of a common dormancy continuum, in which VBNC cells represent the most dormant forms (21–23).

Moreover, although persisters were originally described as dormant (24), recent studies have questioned this state (25–27), especially in intracellular niches, where persisters generally sustain metabolic activity to withstand host-specific stresses (28, 29), pointing to a crucial role of the environment to modulate their metabolism.

In a previous work, we used Staphylococcus aureus expressing inducible green fluorescent protein (GFP) to monitor bacterial division at the single-cell level (30) by following via flow cytometry the decrease in the fluorescence signal upon cell division after removal of the inducer at the end of the phagocytosis period (31). We demonstrated that phagocytic cells host a pool of metabolically active S. aureus cells during antibiotic exposure, which show all the hallmarks of persisters (2), namely, a biphasic rate of killing, a progeny remaining susceptible to the applied antibiotic, a level of persistence weakly dependent on the drug concentration, and an absence of division and lower rate of killing than those observed for the susceptible population from which they emerge (31). Recently, it has been shown that reactive oxygen species (ROS) generated by phagocytes contribute to antibiotic tolerance (11), defined as the capacity of a bacterial cell to survive to antibiotic pressure in the absence of resistance, as objectified by the demonstration of a lower rate of killing (2). Given that oxidative stress promotes numerous types of cellular damage, notably to translation and ATP synthesis (11, 32, 33), we hypothesized that the level of oxidative stress expressed by the host cells could influence the dormancy depth that persisters can reach. The aim of this work was therefore to study the relationship between host cell oxidative stress and dormancy depth of intracellular persisters during antibiotic exposure. In addition, as S. aureus infections may affect various tissues and cell types, we also examined the heterogeneity of persisters’ dormancy in relation to their host niche during exposure to a same antibiotic pressure, but with host cells displaying various oxidative stress levels.

Using single-cell techniques, we found that host oxidative stress drives in a dynamic fashion the formation of dormant states of S. aureus persisters, characterized by increased lag time for resuscitation and regrowth in liquid medium, and is strictly correlated with the level of ROS produced by each host cell. In high-oxidative-stress cells, ROS-induced ATP depletion was associated with the formation of visible dark foci observed during protein aggregation, together with the recruitment of the DnaK-ClpB chaperone system, involved in the clearance of protein aggregates. We also found that ATP depletion alone promotes deeper dormancy than oxidative stress, due to counterbalancing ROS-induced translational repression, and that exposure to a protein synthesis inhibitor limits dormancy to a level similar to that observed in low-oxidative-stress cells. We concluded that both translational and ATP synthesis defects regulate bacterial dormancy. Our work adds to current knowledge that the level of oxidative stress imposed by host cells drives heterogeneous dormancy depths among persister populations, which may have important implications regarding the capacity of S. aureus to persist in different tissues or cell types.

## RESULTS

Our previous work (31) showed that an antibiotic pressure is required to select persisters in permissive cells but that a similar residual fraction of nondividing bacteria is measured in permissive cells (J774 macrophages) exposed to oxacillin at 50× the MIC or in nonpermissive cells (human macrophages) incubated or not with the antibiotic (see Fig. S1 in the supplemental material), indicating that the fraction of persistence is not directly dependent on the type of stress imposed on the bacteria. Here, our aim was to examine the level of dormancy of these persister fractions surviving in cells with different levels of oxidative stress. All cell types were therefore exposed to the same antibiotic pressure (i.e., incubation with 50× the MIC of oxacillin) in order to select the persister subpopulation in all cases.

### Host oxidative stress drives transition to dormant states of S. aureus persisters.

**FIG 1:**
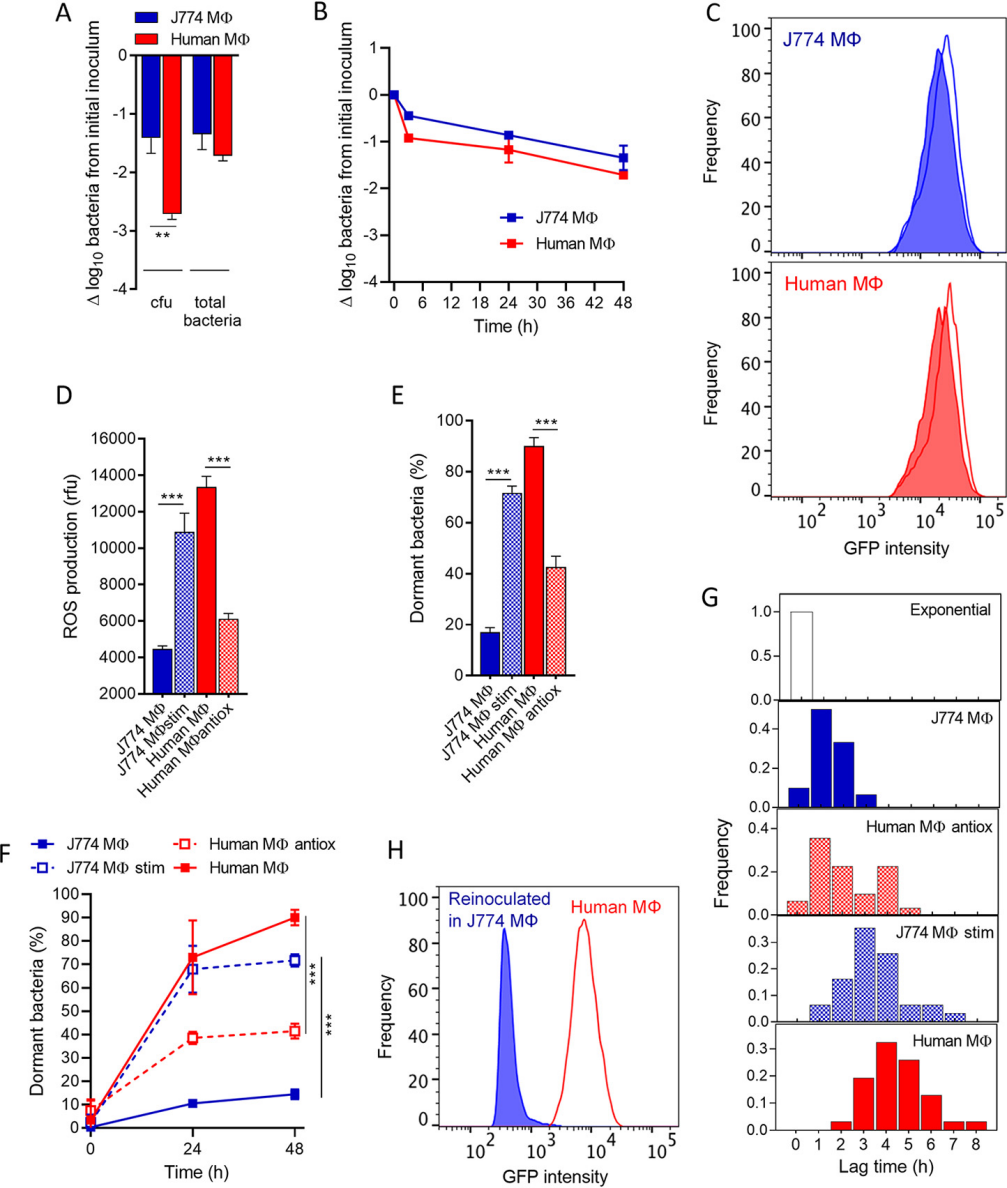
Host oxidative stress drives transition to dormant states of S. aureus persisters. (A) Bacteria (CFU or total propidium iodide-negative bacteria from flow cytometry profiles) recovered from macrophages (MΦ) exposed to 50× the MIC of oxacillin for 48 h. (B) Time-kill curves against S. aureus infecting macrophages exposed to 50× the MIC of oxacillin, displaying total propidium iodide-negative bacteria from flow cytometry profiles. The kill rate is estimated as a 0.2- or 0.3-log_10_ decrease in propidium iodide-negative events per hour over the first 3 h of incubation in J774 and human macrophages, respectively, and a 0.02-log_10_ decrease per hour for longer incubations up to 48 h in both cell types. (C) Flow cytometry profiles of persisters recovered from macrophages exposed to 50× the MIC of oxacillin for 48 h (fill) and their respective postphagocytosis inoculum (line). (D) Cellular ROS production was measured using the oxidation-sensitive fluorescent probe DCF. Cells were incubated with 10 μM DCF for 30 min prior to fluorescence measurement. (E and F) Proportions of dormant persisters recovered from macrophages exposed to 50× the MIC of oxacillin for 48 h (E) or for the indicated periods (F). Dormant bacteria are defined as cells nonproliferating for 24 h on an agar plate in comparison to total propidium iodide-negative bacteria from flow cytometry profiles. (G) Awakening kinetics of persisters recovered from macrophages exposed to 50× the MIC of oxacillin for 48 h and reinoculated in fresh MHB-CA medium, starting from a single FACS event. Lag times were determined by densitometry and calculated in comparison to the awakening time of exponential cultures (*n* = 30). (H) Flow cytometry profiles of persisters recovered from human macrophages exposed to 50× the MIC of oxacillin for 48 h and then reinoculated in J774 macrophages for an additional 24 h (1 mg L^−1^ gentamicin to prevent extracellular contamination). Where indicated, J774 macrophages were stimulated with LPS and IFN-γ (J774 MΦ stim), and human macrophages were incubated with BHA (Human MΦ antiox). Data are means ± standard error of the mean (SEM) (A, B, and D to F) or representative results (C, G, and H) from three independent experiments. Statistical analysis was performed with one-way analysis of variance (ANOVA) with Sidak’s posttest. ***, *P* < 0.001.

After 48 h of incubation of cells infected with GFP-induced S. aureus and exposed to 50× the MIC of oxacillin, we determined the number of remaining bacteria, using in parallel CFU counting on agar plates and enumeration of propidium iodide-negative GFP-expressing bacteria by flow cytometry, respectively. This allowed us to distinguish the fraction of intracellular bacteria that were able to form colonies on agar plates from the total population of potentially viable bacteria (i.e., including bacteria that do not necessarily resume growth on agar plates). Both J774 macrophages and human primary macrophages hosted a viable and antibiotic-tolerant pool of bacteria (Fig. 1A). Although a higher bactericidal effect was observed in human macrophages (lower CFU counts), no significant difference was seen among both cell types when considering the total pool of propidium iodide-negative GFP-expressing bacteria by flow cytometry, suggesting that a part of the inoculum from human macrophages has a too low metabolism to efficiently initiate resuscitation on agar plates (17, 34). As observed in persister populations, time-kill curves displayed a biphasic trend in both macrophages to reach a homogeneous population of persisters (Fig. 1B).

In parallel, the corresponding flow cytometry profiles of the propidium iodide-negative bacteria revealed intracellular pools with a uniform nongrowing state, as indicated by the absence of decrease in GFP signals compared to the postphagocytosis inoculum during 48 h postinfection (Fig. 1C). This nongrowing state is a typical characteristic of persisters (2).

We then analyzed the heterogeneity of these nongrowing populations, especially their ability to resuscitate from their resting state, as an indicator of their dormancy level (17, 27). Before investigating the role of host reactive oxygen species (ROS) in dormancy, we first measured the host cells’ ROS production (Fig. 1D). We did not determine the nature or the site of production of ROS in these cells, but ROS produced via the phagosomal NADPH oxidase machinery are considered to play a prominent role in the fight against intracellular pathogens (35), and intracellular S. aureus is reported to survive in the vacuoles of phagocytes (36)—at least when the bacterium is not replicating (37). Human macrophages were higher ROS producers than J774 macrophages, the latter being known as low-oxidative-stress cells (11). We also stimulated J774 macrophages with lipopolysaccharide (LPS) and gamma interferon (IFN-γ), and treated human macrophages with the antioxidant butylated hydroxyanisole (BHA), resulting in enhanced and decreased ROS production, respectively (38). Both of these derived macrophages hosted nondividing bacteria under our conditions (see Fig. S2 in the supplemental material) that can be defined as persisters.

To study the level of dormancy among the persister populations hosted by these different macrophages, the number of CFU was compared to the total pool of bacteria enumerated from flow cytometry profiles (from which dead cells were excluded based on propidium iodide staining), following the same procedure described above. Data were expressed as the percentage of dormant cells in the bacterial population. Dormant bacteria were defined as bacterial cells detected by flow cytometry, but nonproliferating for 24 h on an agar plate (Fig. 1E). Bacterial pools hosted in J774 macrophages were predominantly able to form colonies, meeting the classic definition of persisters, which recover their growth capacity when the environmental pressure is relieved (2). In contrast, the pool hosted in human macrophages almost exclusively consisted of bacteria meeting the criteria of dormant populations.

Stimulated J774 macrophages hosted a proportion of dormant persisters comparable to that found in human macrophages. Conversely, most persisters collected from BHA-treated human macrophages were able to exit from dormancy on agar plates. These data support the conclusion that ROS production by macrophages can promote dormancy among persisters. Importantly, we found that the dormant fractions rapidly increased during the first 24 h of infection, confirming that dormancy is a dynamic process (Fig. 1F).

To further quantify the dormancy levels, we measured single-cell lag times for regrowth in liquid medium, a parameter considered an experimentally measurable estimation of dormancy levels (17). Persisters recovered from macrophages were reinoculated in fresh cation-adjusted Mueller-Hinton broth (MHB-CA) medium, starting from a single fluorescence-activated cell sorter (FACS) event (Fig. 1G). Among the propidium iodide-negative population recovered by flow cytometry from all phagocytes, we found that 94% of bacteria on average resumed growth spontaneously when reinoculated in liquid medium, thus excluding dead cells and VBNC cells (21, 39). Compared to exponential cultures, we found that persisters recovered from infected cells showed heterogeneous and increased lag times, consistent with lag time modulation after antibiotic challenge in Escherichia coli (40) or after neutrophil challenge in S. aureus (27). Additionally, we observed that persisters hosted in human macrophages started to proliferate even later than those hosted in J774 macrophages. The lag times were also further delayed in persisters hosted in stimulated J774 macrophages and shortened in BHA-treated human macrophages, suggesting either variable levels of metabolism or the need for repair upon wakening. These results were also confirmed at the population level (see Fig. S3 in the supplemental material). We therefore conclude here that host oxidative stress led to deeper dormancy and prolonged lag times among persisters.

Importantly, we found that, despite their dormancy, persisters isolated from human macrophages remained not only viable but also infectious and resumed growth when reinoculated in J774 macrophages without antibiotic challenge, as evidenced by the reduced GFP content after reinoculation (Fig. 1H), thereby confirming the rapid adaptation of persisters to their niche.

### ROS-induced ATP depletion correlates with the formation of dark foci.

**FIG 2:**
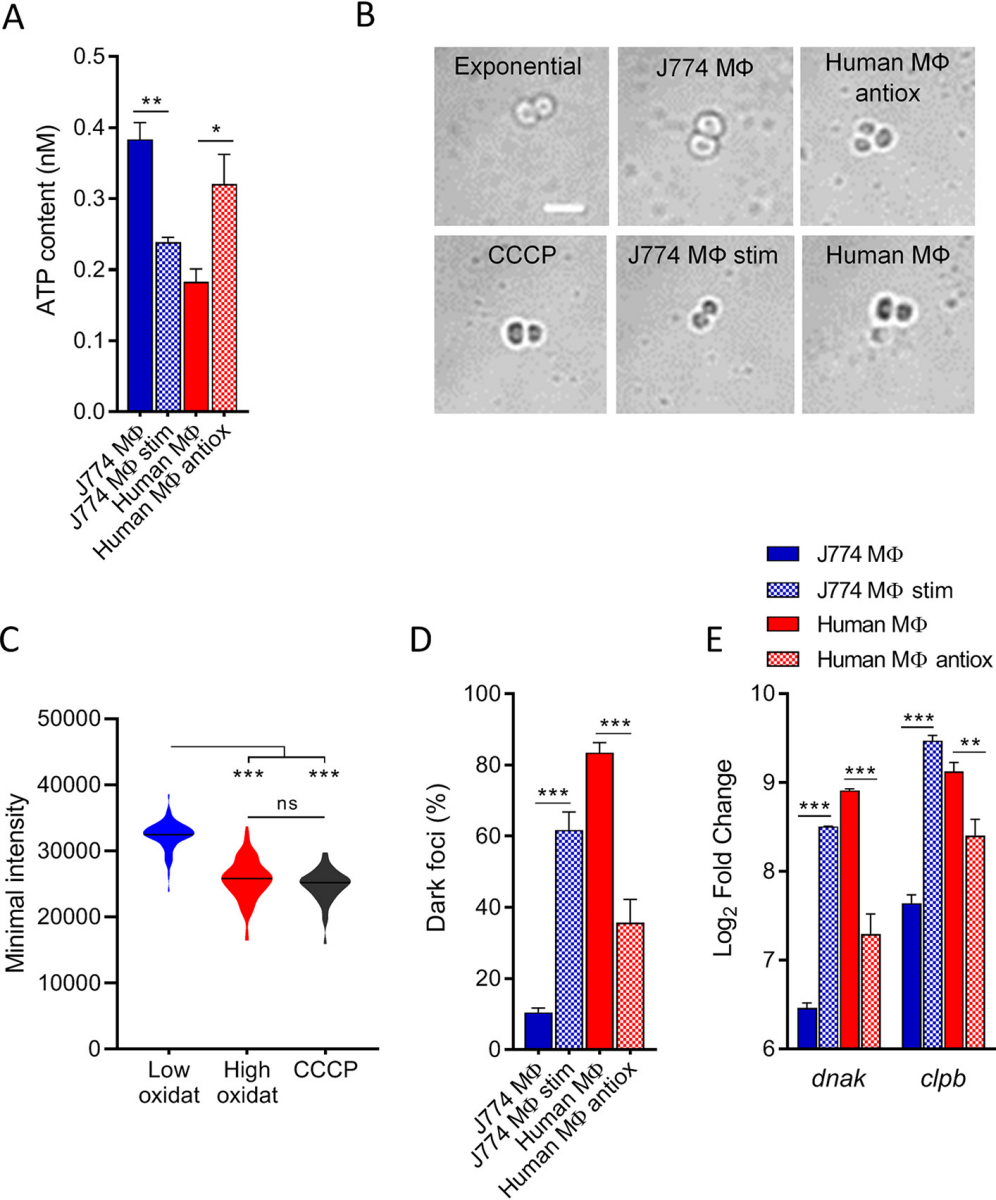
ROS-induced ATP depletion correlates with the formation of dark foci. (A) Intrabacterial ATP concentration of persisters recovered from macrophages exposed to 50× the MIC of oxacillin for 48 h, normalized to the total number of viable bacteria. (B) Bright-field images (confocal microscopy) of persisters recovered from macrophages exposed to 50× the MIC of oxacillin for 48 h, 8-h cultures exposed to CCCP for 24 h, or exponential-phase cultures (scale bar, 2 μm). (C) Violin plots displaying the minimal intensity in the bright-field channel of counted bacteria. Bacteria were recovered from 8-h cultures exposed to 6 μM CCCP for 24 h or from macrophages exposed to 50× the MIC of oxacillin for 48 h. Bacteria from macrophages were classified into two groups based on the level of host cell oxidative stress: low oxidative stress (i.e., J774 MΦ and Human MΦ antiox) and high oxidative stress (i.e., J774 MΦ stim and Human MΦ). For low oxidative stress, *n* = 63, for high oxidative stress, *n* = 94, and for CCCP, *n* = 78. Black lines indicate median values. (D) Proportions of persisters displaying dark foci under confocal microscopy. Persisters were recovered from macrophages exposed to 50× the MIC of oxacillin for 48 h. (E) Quantitative real-time PCR of *dnaK* and *clpB* transcripts in persisters recovered from macrophages exposed to 50× the MIC of oxacillin for 48 h. Where indicated, J774 macrophages were stimulated with LPS and IFN-γ (J774 MΦ stim), and human macrophages were incubated with BHA (Human MΦ antiox). Data are means ± SEM (A and C to E) or representative results (B) from three independent experiments. Statistical analysis was performed by one-way ANOVA with Sidak’s posttest. ***, *P* < 0.001; **, *P* < 0.01; *, *P* < 0.05; ns, not significant.

Oxidative stress is known to promote numerous cellular damages, especially energy metabolism alterations (11). Among them, ATP synthase defects are presumably a consequence of a reorientation of the respiratory chain, the electrons of which are required for reduction of oxidized proteins (41). Accordingly, dormant persisters hosted in high-oxidative-stress cells (i.e., human macrophages and stimulated J774 macrophages) displayed low ATP levels that were brought back to levels observed in J774 macrophages by exposure to antioxidant (Fig. 2A).

ATP plays a central role in maintaining bacterial proteostasis, by preventing protein aggregation through its hydrotropic effects (42) and by fueling ATP-dependent chaperone systems (e.g., DnaK and ClpB) (43). Consequently, ATP depletion has been described to drive protein aggregation, visible as dark foci within the bacterial cells under bright-field microscopy (17). In addition, ROS can also favor protein aggregation by inducing oxidation or other modifications of amino acid residues and by modifying their folding properties (32, 33, 44). We thus investigated the presence of dark foci in persisters hosted by the different macrophages.

Under bright-field microscopy, we observed dark foci, predominantly at the cell periphery, in the majority of persisters hosted by high-oxidative-stress cells, while there were almost absent in persisters hosted by low-oxidative-stress cells, as well as in growing cultures (Fig. 2B). These dark foci were also observed in cultures exposed to CCCP (carbonyl cyanide *m*-chlorophenylhydrazone), a proton motive force inhibitor that corrupts ATP synthase activity and produces protein aggregates (17, 45). To confirm the presence of dark foci, the level of light transmitted by bacterial cells from bright-field images was recorded. The signals from persisters were classified into two groups, based on the level of ROS measured in the cells hosting them, and compared to the light intensity transmitted by CCCP-exposed bacteria. We found a significant decrease in the light intensity for dormant persisters hosted in high-oxidative-cells cells (Fig. 2C), similar to that reached in the presence of CCCP, reflecting the internal complexity appearing in an oxidative environment. Consistently, we found a similar proportion of bacteria containing dark foci (Fig. 2D) and of dormant bacteria (Fig. 1E).

Once protein aggregation occurs, the DnaK-ClpB chaperone system plays a major role in protein homeostasis and clearance of aggregates, which in turn allows growth resumption of dormant bacteria (17, 43, 46). Consistently, we found that d*nak* and c*lpB* were overexpressed in dormant persisters hosted in high-oxidative-stress host cells (Fig. 2E).

During entry into quiescence, protein aggregates are known to be enriched in proteins vital for cellular functions, especially those related to energy metabolism and ribosomal machinery (17, 18, 47). In addition, ATP depletion can result in a decrease transcription of the translation machinery, given that a large part of the cell energy is devoted to protein synthesis (48). Accordingly, we found decreased transcription of typical members of ribosomal machinery (i.e., the *rplA*, *rplM*, and translation factor *prfA* genes) and of energy production (i.e., the *ldhD* [lactate fermentation] and *atpA* [oxidative phosphorylation] genes) in persisters hosted by high-oxidative-stress cells (see Fig. S4 in the supplemental material), confirming an alteration of the corresponding pathways and pointing to a general decline of metabolism in dormant persisters.

Together, these data lead us to conclude that ROS-induced ATP depletion in dormant persisters drives the formation of dark foci observed during protein aggregation.

### ROS-induced translational and ATP synthesis defects regulate bacterial dormancy depth.

Because oxidative stress is known to promote notably translational defects (49), we then investigated the role of translation in the dormant phenotype.

**FIG 3:**
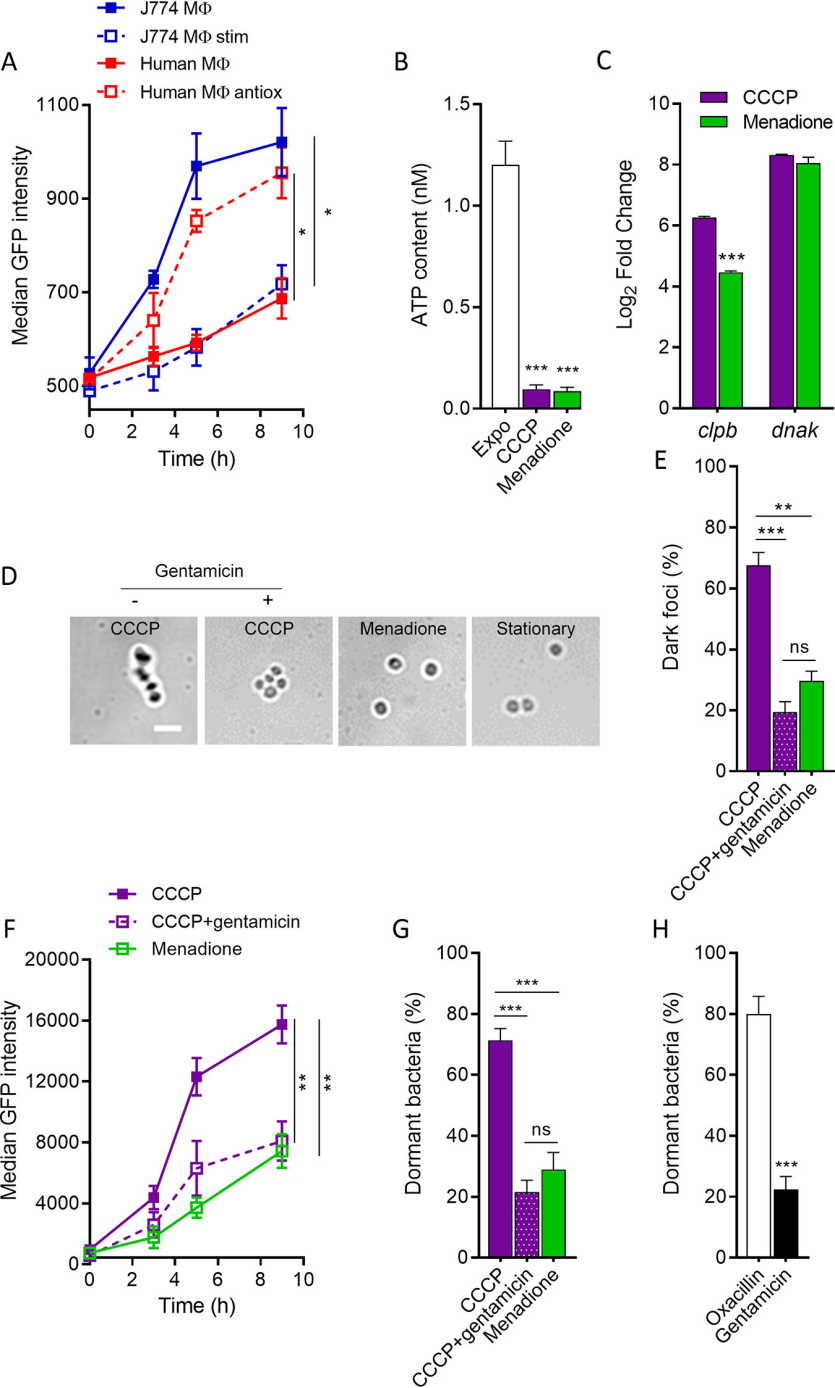
ROS-induced translational and ATP synthesis defects regulate bacterial dormancy depth. (A) Translation rate of intracellular persisters. Macrophages were infected by noninduced bacteria for 48 h with 50× the MIC of oxacillin and then induced for GFP expression for the indicated periods. Where indicated, J774 macrophages were stimulated with LPS and IFN-γ (J774 MΦ stim), and human macrophages were incubated with BHA (Human MΦ antiox). (B) Intrabacterial ATP concentration of exponential-phase cultures and 8-h cultures exposed to 6 μM CCCP or 80 μM menadione for 24 h, normalized to the total number of viable bacteria. (C) Quantitative real-time PCR of *dnaK* and *clpB* transcripts in 8-h cultures exposed to 80 μM menadione or 6 μM CCCP. (D) Bright-field images (confocal microscopy) of 8-h cultures grown under the control condition or exposed to 80 μM menadione or 6 μM CCCP ± 0.5× the MIC of gentamicin (scale bar, 2 μm) and proportions of bacteria displaying dark foci (E). (F) Translation rate of 8-h noninduced cultures exposed to 80 μM menadione or 6 μM CCCP ± 0.5× the MIC of gentamicin. Bacteria were then induced for GFP expression for the indicated periods. (G) Proportions of dormant bacteria in 8-h noninduced cultures exposed to 80 μM menadione or 6 μM CCCP ± 0.5× the MIC of gentamicin. Dormant bacteria are defined as cells nonproliferating for 24 h on an agar plate in comparison to total propidium iodide-negative bacteria from flow cytometry profiles. (H) Proportions of dormant bacteria recovered from stimulated J774 macrophages exposed to 50× the MIC of oxacillin or 50× the MIC of gentamicin for 48 h. Dormant bacteria are defined as cells nonproliferating for 24 h on an agar plate in comparison to propidium iodide-negative bacteria from flow cytometry profiles. Data are means ± SEM (A to C and E to H) or representative results (D) from three independent experiments. Statistical analysis was performed with two-tailed Student's *t* test (H) and one-way ANOVA with Sidak’s posttest for multiple comparison (A to C, F, and G). ***, *P* < 0.001; **, *P* < 0.01; *, *P* < 0.05; ns, not significant.

We first measured the neo-synthesis of GFP in initially noninduced populations of intracellular persisters selected after 48 h of incubation with oxacillin at 50× the MIC (Fig. 3A). Persisters hosted in human macrophages (essentially corresponding to dormant bacteria) poorly responded to induction in comparison with their J774 macrophage counterparts (mainly corresponding to nondormant bacteria). Stimulation of J774 macrophages reduced translation levels to a level similar to that observed in human macrophages, which was in turn restored by the addition of an antioxidant, thereby confirming the role of ROS in inducing translational defects. Of note, these translational defects cannot be ascribed to a global inhibition of transcription (Fig. 2E; see Fig. S4 to S6 in the supplemental material).

We then investigated the role of such translational defects under conditions leading to dormancy (i.e., ATP depletion). We treated entry of stationary-phase cultures (8 h) for 24 h with either CCCP (i.e., an inhibitor of ATP synthesis) or menadione (i.e., an inducer of ROS production via redox cycling) to mimic the oxidative stress faced by the bacteria in the phagocytic environment. At concentrations of 6 μM CCCP and 80 μM menadione, both compounds led to a similar drop in ATP content (Fig. 3B). However, CCCP led to a more important expression of *clpB* chaperone than menadione (Fig. 3C) and also significantly higher proportions of bacteria with dark foci than menadione (Fig. 3D and E), suggesting that these effects are not directly related to the ATP content.

As an ongoing translation is required for protein aggregation (50), inhibition of translation has been shown to limit aggresome formation in E. coli (17). To test if the same mechanism applies under our conditions, we incubated CCCP-treated cultures with 0.5× the MIC of gentamicin (i.e., a protein synthesis inhibitor). These bacteria contained few visible dark foci (Fig. 3D), as observed in menadione-treated cultures (Fig. 3E), and their translation level was decreased to a level similar to that observed in menadione-treated cultures (Fig. 3F). The presence of protein aggregates was corroborated by SDS-PAGE for bacteria incubated with CCCP or menadione (Fig. S5), confirming that bacteria displaying dark foci are enriched in insoluble proteins by a process of ATP-dependent dynamic protein aggregation (17).

We likewise found that CCCP-treated cultures display larger proportions of dormant bacteria than menadione-treated cultures and that gentamicin treatment prevents dormancy development (Fig. 3G). These data further confirm the link between dark focus formation and dormancy and indicate that inhibition of protein synthesis limits protein aggregation in dormant bacteria.

In line with these observations, we then exposed high-oxidative-stress cells (i.e., stimulated J774 macrophages) to 50× the MIC of oxacillin or gentamicin for 48 h. We found that exposure to the protein synthesis inhibitor limits dormancy of intracellular persisters to levels comparable to those observed in low-oxidative-stress cells (Fig. 3H). Collectively, this indicates that ROS-induced dormancy develops as a consequence of the balance between ROS-induced ATP depletion and translation defects and confirms the central role of translation in protein aggregation and consecutive dormancy.

### Host oxidative stress drives heterogeneous dormancy of S. aureus persisters among host cells.

**FIG 4:**
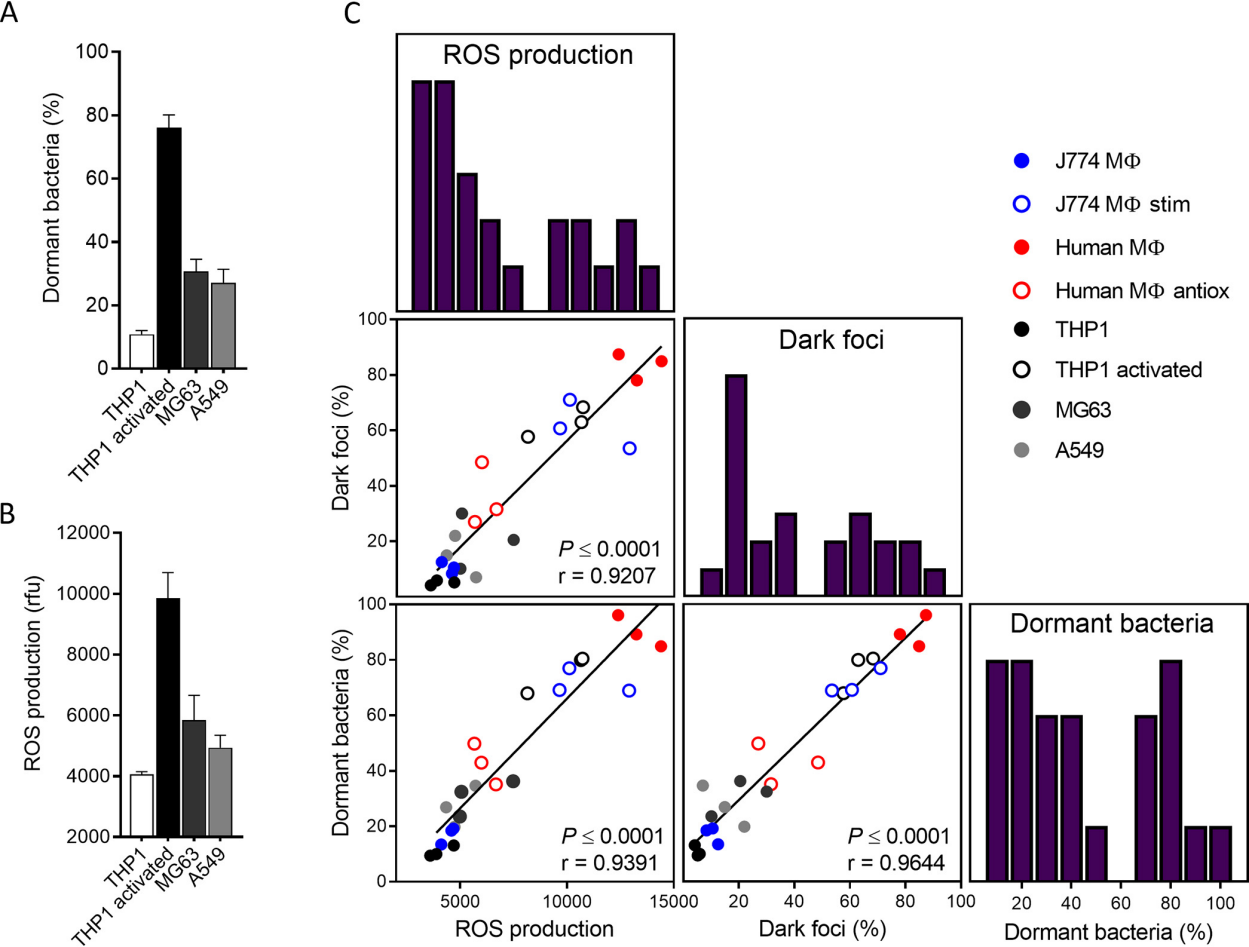
Host oxidative stress drives heterogeneous dormancy of S. aureus persisters among host cells. (A) Cellular ROS production was measured using the oxidation-sensitive fluorescent probe DCF. Cells were incubated with 10 μM DCF for 30 min prior to fluorescence measurement. (B) Proportions of dormant persisters recovered from different host cell types exposed to 50× the MIC of oxacillin for 48 h. Dormant bacteria are defined as cells nonproliferating for 24 h on agar plates in comparison to total propidium iodide-negative bacteria from flow cytometry profiles. (C) Correlation matrix between ROS production, proportions of bacteria displaying dark foci and proportions of dormant bacteria, and their respective frequency distributions. Pearson correlation coefficients (*r*) and significance (*P*) are provided for each correlation. Where indicated, THP-1 monocytes were incubated with 200 ng mL^−1^ PMA for 48 h (THP-1 activated). Data are means ± SEM from three independent experiments.

To strengthen our findings on the implication of host ROS in the dormant phenotype of persisters, we then extended our experiments to other cell types, namely, human THP-1 monocytes and THP-1 cells pretreated with phorbol 12-myristate 13-acetate (PMA) (which results in increased phagocytic activity and superoxide production [51, 52]), as well as nonprofessional phagocytes (i.e., human epithelial cells of the A549 line and osteoblasts of the MG63 line). We found that monocytes, epithelial cells, and osteoblasts were low ROS producers (Fig. 4A) and hosted a limited fraction of dormant persisters (Fig. 4B). In contrast, PMA-treated monocytes displayed enhanced ROS production, which correlates with massive increase in dormant fractions, pointing to a causal role of oxidative stress in dormancy.

Collectively, the data obtained in these different cells combined with those collected in the different macrophages highlight strong correlations between (i) the levels of host oxidative stress, (ii) the formation of dark foci, and (iii) the emergence of dormant persisters (Fig. 4C). This led us to conclude that host oxidative stress is the major force driving ATP depletion, which in turn can contribute to promote dark focus formation, the subsequent dormancy state, and increased lag times, leading to highly heterogeneous dormancy depth among persister populations.

## DISCUSSION

While persistence and its related quiescence are mostly studied under nutrient stress conditions (18, 53, 54), we study here persisters’ dormancy in intracellular compartments, which results in higher persister fractions than those generally reported in planktonic cultures during antibiotic challenge (2, 55, 56). Our work highlights how the level of oxidative stress from the intracellular environment can modulate dormancy among populations of S. aureus persisters, leading to additional increases in lag times and diversification during bacterial awakening. Our observations therefore support the hypothesis that intracellular S. aureus persisters can reach different dormancy depth depending on their host cells.

**FIG 5:**
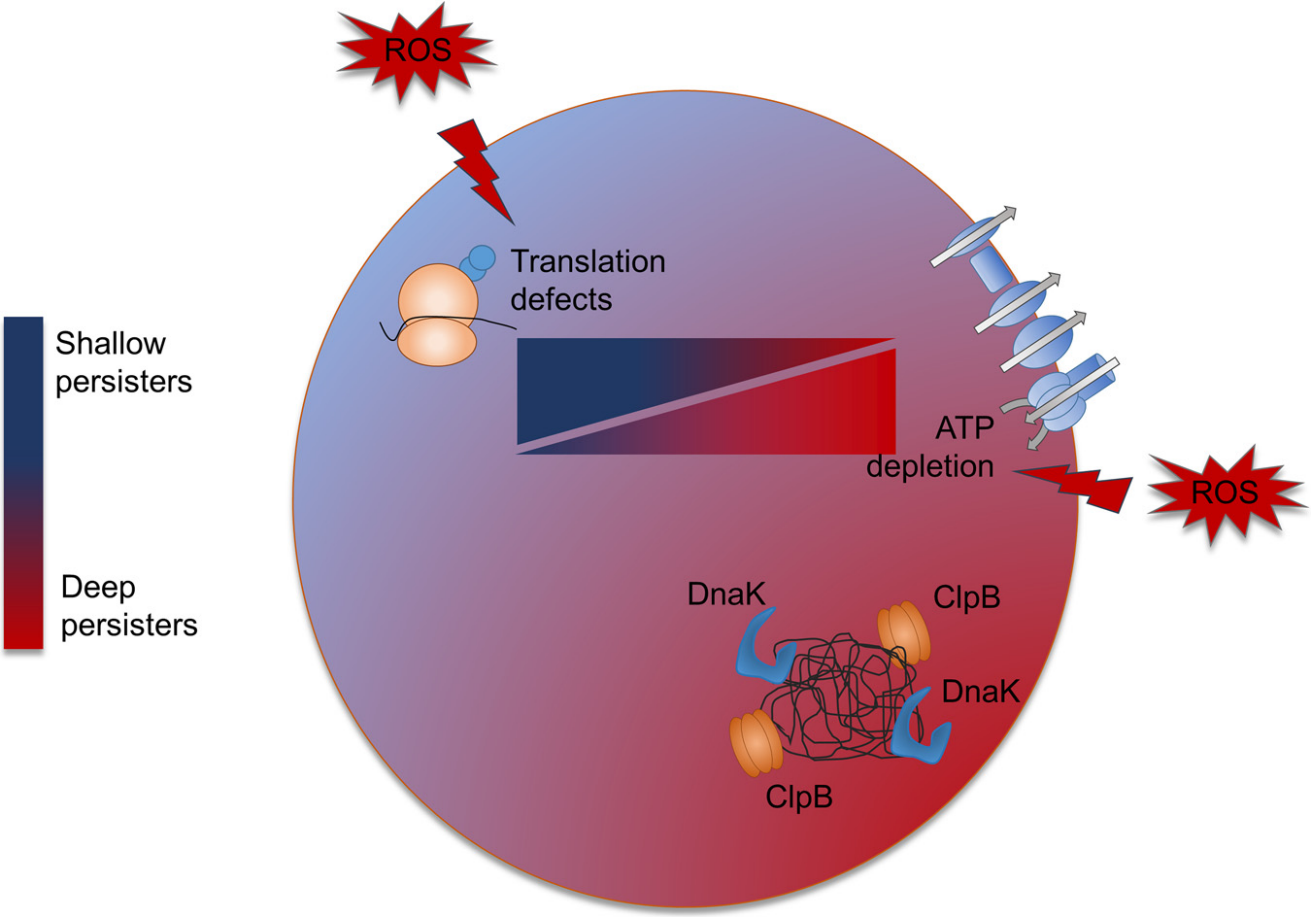
Model for dormancy of S. aureus persisters under host oxidative stress. In low-oxidative-stress cells, persisters do not show signs of the dark foci observed during protein aggregation. In an oxidative host environment, persisters face ROS-induced translational and ATP synthesis defects; the resulting balance between them defines the dormancy depth. ATP depletion induces protein aggregates that bacteria clear prior to growth resumption, resulting in increased lag times. On the other hand, translation plays a pivotal role to fine-tune the deepness of dormancy, and translation inhibition decreases protein aggregation, resulting in reduced dormant fractions.

Collectively, we propose a model for dormancy of S. aureus persisters driven by host oxidative stress (Fig. 5). In an oxidative host environment, S. aureus persisters face ROS-induced translational and ATP synthesis defects, both of which regulate bacterial dormancy depth. ATP depletion is associated with the formation of visible dark foci observed during protein aggregation, which strictly correlates with the level of oxidative stress. Growth resumption relies on both ATP replenishment and clearance of protein aggregates through the ATP-dependent DnaK-ClpB system, resulting in prolonged lag times after stress removal (11). Besides ATP depletion, our data indicate that formation of dark foci is attenuated by ROS-induced translational defects, confirming a central role of translation in dormancy (18, 57, 58).

Lag time diversification resulting from heterogeneous dormancy depths might constitute a “bet-hedging” strategy that favors bacterial survival in fluctuating host environments (34, 59). As the clinical meaning of dormant S. aureus cells and their evolution to VBNC is still unclear, further experiments will be required to determine whether it is more beneficial to favor bacterial aggregation to a level where bacteria will no longer revert to growing phenotypes or, the opposite, help bacteria awaken (60–62). In the last case, the model we propose here supports the hypothesis that either inhibiting translation or forcing bacteria to maintain regular energy metabolism could prevent ROS-induced dormancy of S. aureus.

In spite of this dormancy, all persisters within the different host cells still actively cope with stresses jointly imposed by the cell and the antibiotic, by developing a network of active responses, such as the cell wall stress and SOS responses (i.e., signaling pathways against cell wall and DNA defects, respectively) (Fig. S6). Stress responses appear to be an invariant in the persister phenotype and do not seem to be antagonistic with dormancy (31). Taken together, these findings tend to reconcile conflicting observations about the metabolic status of persisters and to confirm that dormancy should be considered a dynamic continuum (21, 23), the level of which is directly correlated with the sensed level of environmental stress. This continuum likely displays two extremes that are both antibiotic tolerant: persisters that easily escape from dormancy (i.e., shallow persisters) and VBNC cells that are too dormant to resume growth in conventional media. Conceptually, the dormant persisters described in this study may represent an intermediate state between these two extremes, with increased but still measurable lag times.

Importantly, S. aureus persisters can evolve to dormant forms, but can still resuscitate from their resting state within host cells. This leads us to hypothesize that dormant persisters could also participate in recalcitrant infections, by creating long-term and silent niches for persistence.

Finally, dormancy also appears as a dynamic phenomenon that needs to be considered in experimental procedures. Our work calls for caution when analyzing data based on conventional detection methods without the support of single-cell analyses, which could critically influence the way persistence and host-pathogen interactions are interpreted.

## MATERIALS AND METHODS

### Bacterial strain and cells.

The S. aureus susceptible strain SH1000 was transformed to harbor the pALC2084 plasmid containing a tetracycline-inducible *gfp* gene (63). GFP-expressing S. aureus was used for fluorescence dilution experiments as a bacterial division reporter, by monitoring with flow cytometry the decrease in GFP signal after removal of the inducer (31). GFP production was induced by a subinhibitory concentration (125 ng mL^−1^) of tetracycline, and bacteria were routinely grown at 37°C in cation-adjusted Mueller-Hinton broth (MHB-CA) (Sigma) under shaking at 300 rpm.

Murine J774A.1 macrophages (Sandoz Forschung Laboratories) and human macrophages were used for experiments. Human macrophages were obtained by differentiation of monocytes from peripheral blood (64). Briefly, monocytes were isolated from buffy coats of healthy blood donors by a double Ficoll and Percoll density gradient and differentiated in macrophages in RPMI 1640 with 2% human serum (Biowest), 1% penicillin–streptomycin (Thermo Fisher Scientific), and 2.5 ng mL^−1^ macrophage colony-stimulating factor (M-CSF) (Miltenyl Biotec) for 7 days. Macrophages were seeded in 12-well plates (Greiner Bio-One) and incubated with RPMI 1640 medium (Thermo Fisher Scientific) supplemented with 10% fetal bovine serum (FBS) (Thermo Fisher Scientific) at 37°C in a 5% CO_2_ atmosphere. Where indicated, J774A.1 cells were stimulated with RPMI supplemented with 500 ng mL^−1^ LPS from Escherichia coli O55:B5 (Sigma) and 20 ng mL^−1^ recombinant murine IFN-γ (Peprotech) for 20 h before infection, and human macrophages were incubated with 20 μM butylated hydroxyanisole (BHA) (Sigma) during infection (38). Human THP-1 monocytes (ATCC TIB-202) were cultured in RPMI 1640 medium with 10% FBS as previously described (65). Where indicated, THP-1 monocytes were differentiated into adherent macrophages (referred to here as activated THP-1) by incubation with 200 ng mL^−1^ phorbol 12-myristate 13-acetate (PMA; Sigma) for 48 h before infection. Human MG63 osteoblastic cells (LGC Standards) were cultured in Dulbecco’s modified Eagle’s medium (DMEM) with 10% FBS (66), and human A549 epithelial cells (ATCC CCL-185) were cultured in DMEM with 10% FBS.

### Cell infection.

Infections were performed as previously described (31). S. aureus cells were grown overnight and induced for GFP production and resuspended in RPMI 1640 with 125 ng mL^−1^ tetracycline and 10% human serum to allow opsonization for 30 min. Bacteria were incubated with macrophages for 30 min at a multiplicity of infection of 10:1 to allow phagocytosis. Nonphagocytized bacteria were eliminated by a 40-min incubation in RPMI 1640 with 50 mg L^−1^ gentamicin (Sigma). Infected cells were then incubated in RPMI 1640 supplemented with 10% FBS with 50× the MIC of oxacillin (25 mg L^−1^; Sigma) for the indicated periods. Cells were lysed with PBS containing 0.1% Triton X-100 (Sigma). Lysates were centrifuged at 300 × *g* for 5 min to discard cellular debris, and bacteria were collected by centrifugation at 5,000 × *g* for 5 min and processed for CFU counting, flow cytometry, ATP measurements, confocal microscopy, or RNA extraction. For CFU counting, samples were diluted in PBS before plating on agar plates. Data are expressed as log_10_ bacteria per mg cell protein after the incubation period compared to the postphagocytosis inoculum.

### Broth experiments.

The SH1000 strain was grown at 37°C in cation-adjusted Mueller-Hinton broth with 125 ng mL^−1^ tetracycline under shaking at 300 rpm for 8 h. Where indicated, the culture was then exposed to 80 μM menadione (Sigma), 6 μM carbonyl cyanide 3-chlorophenylhydrazone (CCCP) (Sigma), or 0.25 mg L^−1^ gentamicin (0.5× the MIC; Sigma) for 24 h prior to CFU counting, flow cytometry, ATP measurements, bright-filter-mode microscopy, SDS-PAGE, or RNA extraction. For single-cell awakening experiments, persisters recovered from macrophages were reinoculated in fresh MHB-CA medium, starting from of a single FACS event. Lag times were calculated in comparison with the time needed to reach the detection limit in exponential cultures, using a DEN-1 densitometer (Biosan).

### Flow cytometry.

S. aureus cells from planktonic cultures or recovered from macrophages were resuspended in filtered PBS, stained with 10 μg mL^−1^ propidium iodide, and analyzed using a FACSVerse cytometer (BD Biosciences) for GFP signal intensities (fluorescein isothiocyanate [FITC] channel). Forward-scatter width (FCS-W) versus forward-scatter area (FSC-A) and side-scatter width (SSC-W) versus side-scatter area (SSC-A) were used to gate out damaged cells. GFP-positive and propidium iodide-negative events were used for flow cytometry profiles and bacterial counts (31). The number of CFU was subtracted from this total bacterial count to determine the number of dormant bacteria (i.e., cells nonproliferating on an agar plate). Data were analyzed with FlowJo 10.5.2 software (TreeStar, Inc.). The graphs display flow cytometry profiles of the frequency of events as a function of GFP intensity. The number of generations (Fig. S2), *N*, was deduced from *F* = 2*^N^* (67), where the level of replication of the population (fold replication [*F*]) was calculated by the ratio Me_0_/Me*_t_* (Me being the median GFP intensity of the bacterial population at a given time).

### Isolation of insoluble cytosolic proteins SDS-PAGE.

For isolation of insoluble cytosolic proteins by SDS-PAGE, we used a protocol previously described in detail (27), with a few adaptations that are described here. First, at the end of the incubation period, all samples were adjusted to reach approximately 10^10^ CFU mL-1. Second, bacterial lysis was achieved by incubation for 30 min in an adapted buffer B containing 13 CFU mL-1 lysozyme (Sigma) and 130 μg/mL-1 lysostaphin (Sigma) followed by sonication on ice.

### Translation rate.

Macrophages were infected by noninduced bacteria following the procedure described above and incubated with 50× the MIC of oxacillin for 48 h in order to select the population of persisters, after which induction was performed with 125 ng mL^−1^ tetracycline for the indicated periods, and GFP production was followed by measure of the fluorescence signal from flow cytometry profiles (31).

### Confocal microscopy.

Aggregates of proteins cause an important absorbance of light and can be observed as dark foci in bright-field (or phase-contrast) images (68, 69). S. aureus cells from planktonic cultures or recovered from macrophages were fixed with 3.7% paraformaldehyde for 5 min. Bacteria were then washed twice with phosphate-buffered (PBS) and resuspended in water. Prior to microscopy, samples were placed on slides with Dako fluorescence mounting medium (Dako) and covered with cover glasses. Bright-field imaging was performed on a confocal microscope ZEISS LSM 800 (Carl Zeiss) and analyzed with Zen v1.1.2.0 software (Carl Zeiss). Recorded images were analyzed to enumerate the number of bacteria displaying visible dark foci or to measure the minimal light intensity in the bright-field channel of each counted bacterium.

### Quantitative real-time PCR.

S. aureus cells from planktonic cultures or recovered from macrophages were lysed in Tris-EDTA buffer with 13 mg mL^−1^ lysozyme and 130 μg mL^−1^ lysostaphin (Sigma) for 30 min at room temperature. The resulting suspensions were processed for total RNA extraction with the RNA extraction InviTrap Spin Universal RNA minikit (Stratec) and treated with Turbo DNase (Ambion) for 30 min at 37°C, following in both cases the manufacturer’s instructions. Total RNA was reverse transcribed and amplified using a transcription first-strand cDNA synthesis kit (Roche Applied Science). Amplification reactions were performed with SYBR green IQ SuperMix (Bio-Rad Laboratories) with an iCycler iQ single-color real-time PCR detection system (Bio-Rad Laboratories). Primers are listed in Table S1 in the supplemental material. Fold changes in expression versus the control condition were determined using the threshold cycle (2^−ΔΔ^*^CT^*) method, with *gmk* as a housekeeping gene. Control samples were collected from an overnight culture (MHB-CA with 125 ng mL^−1^ tetracycline), centrifuged at 5,000 × *g* for 5 min, resuspended in RPMI 1640, and incubated for 30 min. This bacterial suspension was then mixed with a cell lysate obtained from noninfected macrophages.

### ROS measurements.

Cellular ROS production was measured by using the oxidation-sensitive fluorescent probe 2′,7′-dichlorofluorescin diacetate (DCF) (Sigma) (70). Cells in 12-well plates were washed and resuspended in Hanks' balanced salt solution (Gibco) and incubated with 10 μM DCF for 30 min at 37°C. Fluorescence (excitation at 485 nm and emission at 530 nm) was recorded using a SpectraMax M3 548 microplate reader (Molecular Devices) and normalized to cell content for comparisons.

### ATP measurements.

S. aureus cells from planktonic cultures or recovered from macrophages were washed in 50 mM Tris-HCl, centrifuged at 5,000 × *g* for 5 min, and lysed following the same procedure as for RNA extraction. Lysates were incubated 2 min at 100°C, centrifuged at 9,600 × *g* for 2 min, and assayed for ATP measurements, using the ATP determination kit (Thermo Fisher Scientific) following the manufacturer’s instructions. Bioluminescence measurements were recorded with a SpectraMax M3 548 microplate reader (Molecular Devices) and normalized to the total number of viable bacteria.

### Ethics.

Experiments on blood material were approved by the ethical committee Comité d’Ethique Hospitalo-Facultaire Saint-Luc (permit no B403201730810). Human blood was collected in Croix-Rouge de Belgique centers, from healthy volunteers who gave written informed consent, in accordance with procedures of Service Francophone du Sang de la Croix–Rouge de Belgique.

### Data availability.

Data are available from the corresponding author upon request.
