# Supplementary material for: Host Cell Oxidative Stress Induces Dormant Staphylococcus aureus Persisters
Source: Microbiol Spectr. 2022 Feb 23;10(1):e02313-21. doi: 10.1128/spectrum.02313-21 (PMC8865412; doi:10.1128/spectrum.02313-21)
Supplement: SUPPLEMENTAL FILE 1 — Supplemental material. Download SPECTRUM02313-21_Supp_1_seq2.pdf, PDF file, 0.4 MB [file spectrum02313-21_supp_1_seq2.pdf]

Supplementary information

Host cell oxidative stress induces dormant *Staphylococcus aureus* persisters

Frédéric Peyrusson [1], Tiep Khac Nguyen [1], Tome Najdovski [2], Françoise Van Bambeke [1]

[1] Pharmacologie cellulaire et moléculaire, Louvain Drug Research Institute, Université catholique de Louvain (UCLouvain), Brussels, Belgium, [2] Croix-Rouge de Belgique, Suarlée, Belgium.

This file contains:

Supplementary Figures 1 to 6

Supplementary Table 1

Supplementary Figures

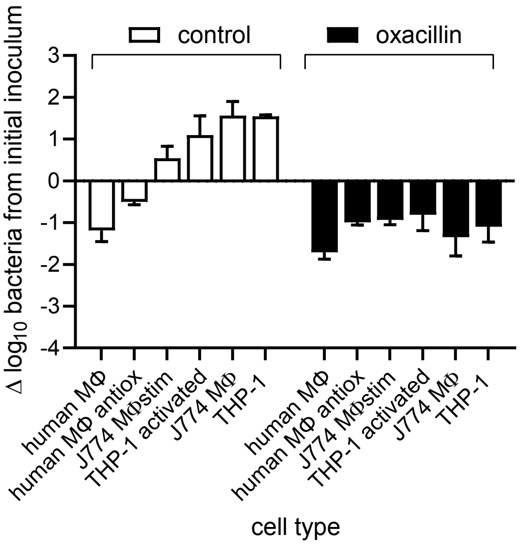

Supplementary Figure 1: Total number of bacteria (propidium-negative events from flow cytometry profiles) recovered from different host cell types exposed to 50 x MIC oxacillin or no antibiotic (gentamicin at its MIC to prevent extracellular contamination) during 48 h. The graph shows that while the rate of bacteria growth or killing is highly variable among cell types in control conditions, the addition of oxacillin allows to reach at least 1 log killing in all cases.

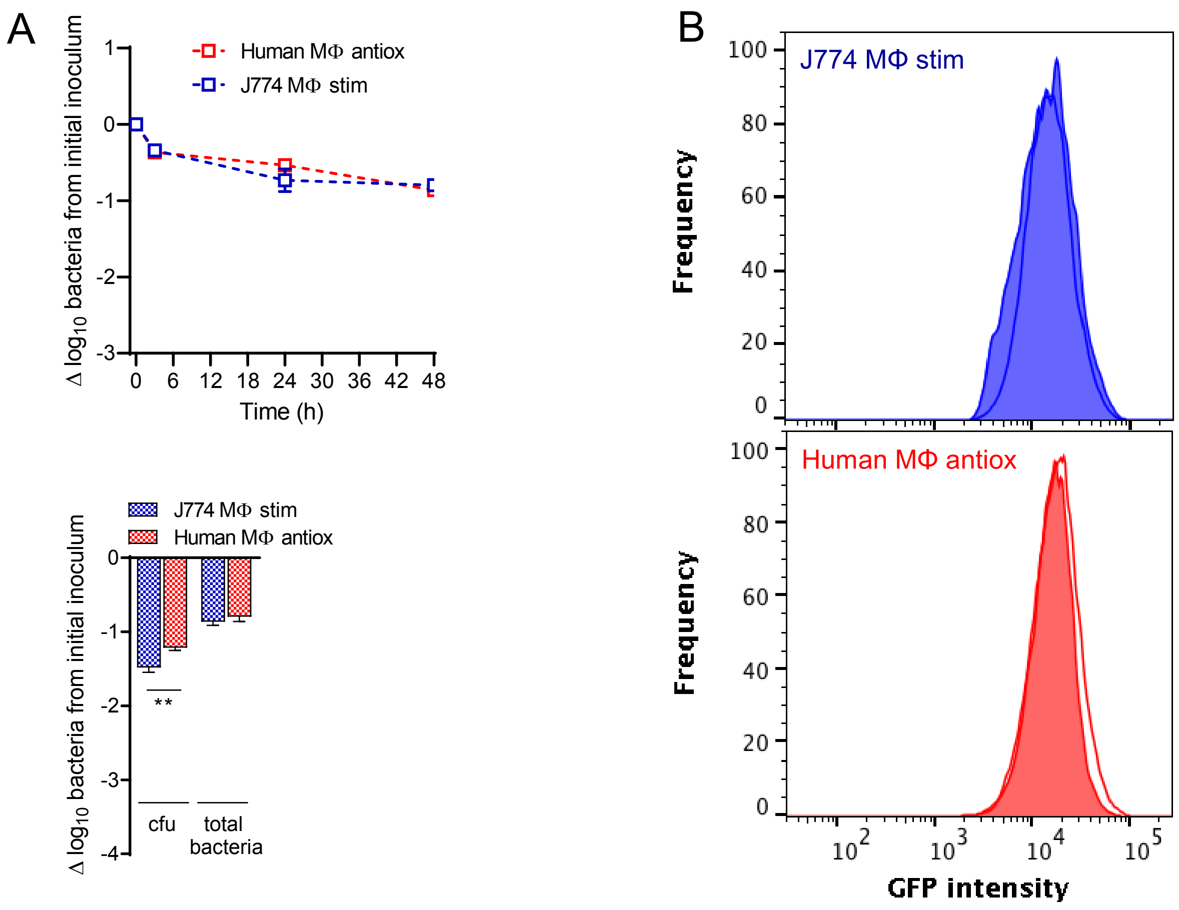

Supplementary Figure 2. Persister formation in stimulated J774 macrophages and antioxidant-treated human macrophages.(A) Top: Time-kill curves against *S. aureus* infecting cells exposed to 50 x MIC oxacillin, displaying total propidium-iodide negative bacteria recorded in flow cytometry profiles. Bottom: Bacteria (cfu or total propidium-negative events in flow cytometry profiles) recovered from macrophages exposed to 50 x MIC oxacillin for 48 h. (B) Flow cytometry profiles of persisters recovered from macrophages exposed to 50 x MIC oxacillin for 48 h (fill), and their respective post-phagocytosis inoculum (line). The graph shows flow cytometry profiles of the frequency of events as a function of GFP intensity. J774 macrophages were stimulated with LPS and IFNγ (J774 MΦ stim), and human macrophages were incubated with BHA (Human MΦ antiox). Data are means ± SEM (A) or representative results (B) from three independent experiments.

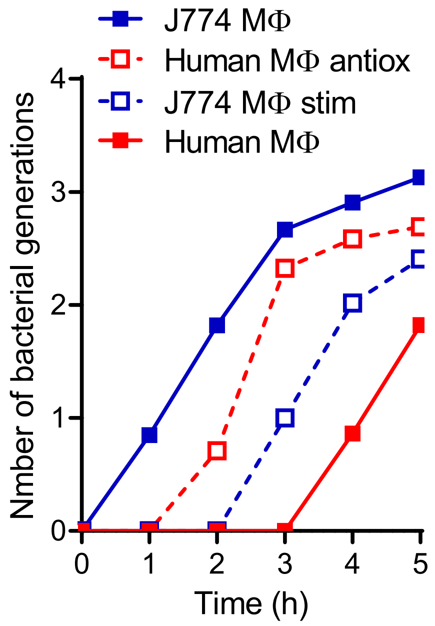

Supplementary Figure 3. Awakening kinetics of persisters in liquid media. Persisters were recovered from macrophages exposed to 50 x MIC oxacillin for 48 h, and reinoculated in fresh MHB-CA medium, starting from an inoculum of 50 single FACS events. Bacterial replication was determined based on cytometry profiles (see Methods). Data are representative results from three independent experiments. Where indicated, J774 macrophages were stimulated with LPS and IFNγ (J774 MΦ stim), and human macrophages were incubated with BHA (Human MΦ antiox).

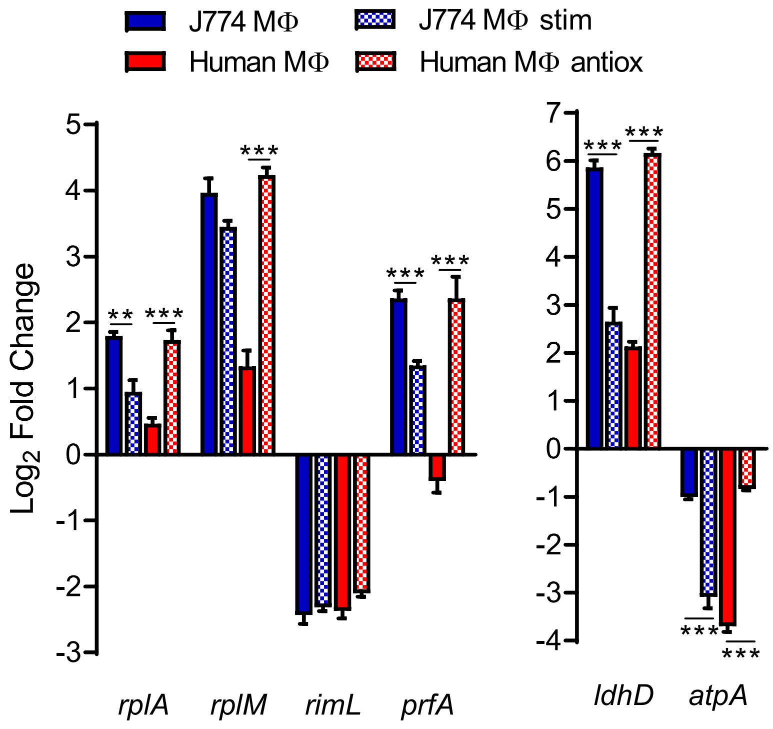

Supplementary Figure 4. Quantitative real-time PCR of transcripts of genes related to translation (left) and energy metabolism (right) in persisters recovered from macrophages exposed to 50 x MIC oxacillin for 48 h. Where indicated, J774 macrophages were stimulated with LPS and IFNγ (J774 MΦ stim), and human macrophages were incubated with BHA (Human MΦ antiox). Data are means ± SEM from three independent experiments. Statistical analysis was performed with one-way ANOVA with Sidak's post-test. ***, $P < 0.001$; **, $P < 0.01$.

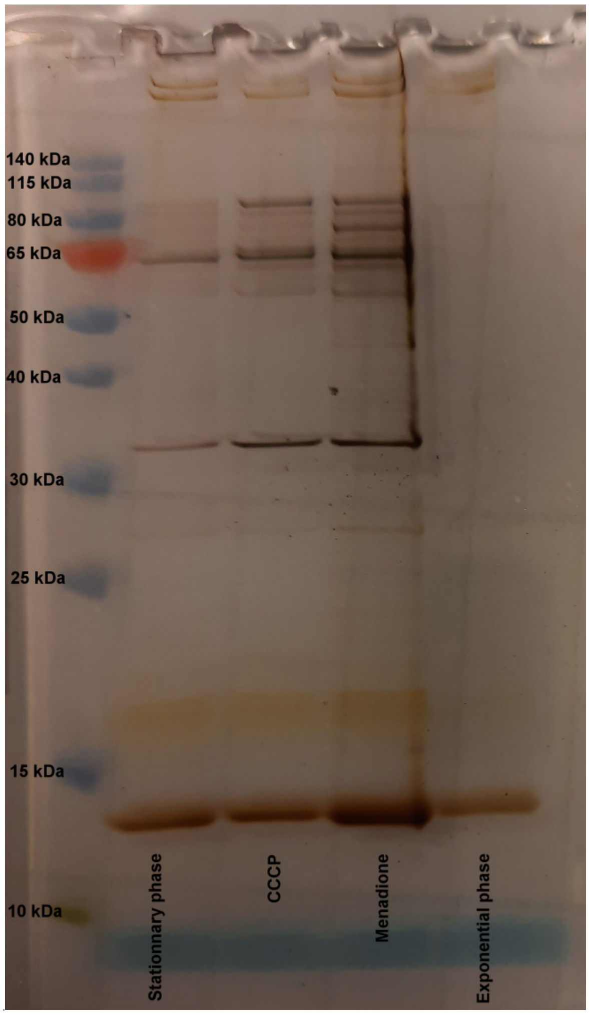

Supplementary Figure 5. SDS-Page of insoluble proteins collected from stationary phase cultures, 8 h cultures incubated with 6 µM CCCP or 80 µM menadione for 24 h, or exponential phase cultures. Each sample was adjusted to a similar number of bacteria (approx. 10^{10} CFUs/mL) before being processed and a same volume was loaded on the gel. High molecular weight proteins were observed in stationary phase cultures as well as in bacteria exposed to CCCP or menadione, but not in exponential phase cultures (known to contain low levels of aggregated proteins), confirming the presence of protein aggregates in the first three conditions.

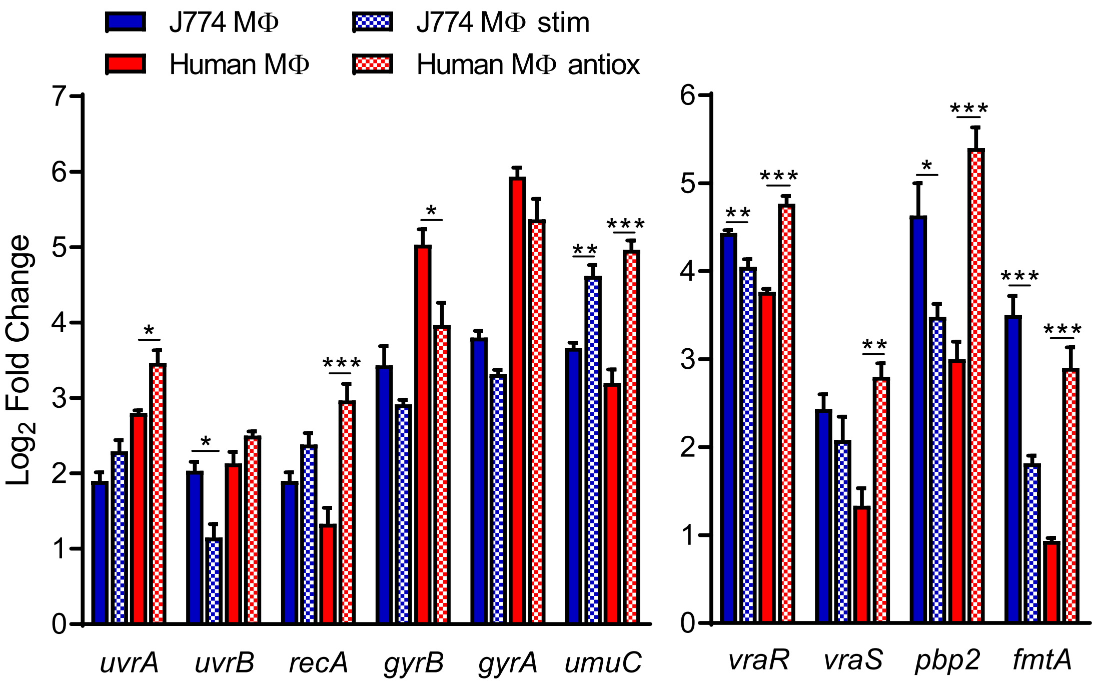

Supplementary Figure 6. Quantitative real-time PCR of transcripts of genes related to SOS (left) and cell-wall stress stimulon (right) in persisters recovered from macrophages exposed to 50 x MIC oxacillin for 48 h. Where indicated, J774 macrophages were stimulated with LPS and IFNγ (J774 MΦ stim), and human macrophages were incubated with BHA (Human MΦ antiox). Data are means ± SEM from three independent experiments. Statistical analysis was performed with one-way ANOVA with Sidak's post-test. ***, $P < 0.001$; **, $P < 0.01$; *, $P < 0.05$.

Supplementary Table 1 Primers for quantitative real-time PCR

Description	Template	Sequence (forward)	Sequence (reverse)
clpB	SH1000	ATGGCCGCGACTTAGTAGAA	ACCAATGAGCACAGGGTTGT
dnaK	SH1000	CGTTTGAAAGATGCTGCTGA	CCGTTTTCACCAGCTGAGAT
gmk	SH1000	AAGGTGCAAAGCAAGTTAGAA	CTTTACGCGCTTCGTTAATAC
rplA	SH1000	CACCAGACATGATGGGTGAA	ACCAGCTTTTTCAGCACGAT
rplM	SH1000	GATGCTGAAGGCCAAACATT	CCAGTATCAACGTGTGGTGTG
rimL	SH1000	CACAAGCAGTTGAGGCATTG	AATCCCAGCCTTTCAGGAAT
prfA	SH1000	GCGATTTTTGCTGGTGATTT	CCACCATGGTCACTTTCAGA
ldhD	SH1000	ACAATTAGTGCGTCGCTTCC	CTGTAGCAGCACCGATACGA
atpA	SH1000	CAAGCAGCAGCTTATCGTGA	CAGTAATTGAACCGCCACCT
uvrA	SH1000	CTCGTGCGCATAACTTGAAA	CGTCGTTGTCCTTCAGCATA
uvrb	SH1000	AATATTCCCAGCCTCTAAAGAAGAA	CTCATCTCGTAATTCTTTCAATCGT
recA	SH1000	GTAGCGCTTCACGCTATTGCT	TTCAAGACCTTGTTCACCATGATC
gyrB	SH1000	GGTGCTGGGCAAATACAAGT	TCCCACACTAAATGGTGCAA
gyrA	SH1000	TCGTGAAGGTGACGAAGTTG	CTGGCGTACGTTTACCATAAC
umuC	SH1000	TGCGAGTGTTTCTTGTATTG	CCCTGTCTTGATGCCTAA
vraR	SH1000	AAAAGATATCGCCGATGCAG	ATAACTCTGCGCGCTTTTTC
vraS	SH1000	AGCTGCATTTCTGTTCATCG	GCGAGTACCGAACCAACAAT
pbp2	SH1000	GGCCATGGTGTATCTGGAGT	GCCGTTAATCCACACGTCTT
fmtA	SH1000	CATCGATTACAGACGAAGACACA	GGCGCAACCTTTTCCTTATT
